# Second law analysis for nonlinear convective flow of a reactive couple stress fluid through a vertical channel

**DOI:** 10.1016/j.heliyon.2018.e00907

**Published:** 2018-11-08

**Authors:** Samuel O. Adesanya, H.A. Ogunseye, R.S. Lebelo, K.C. Moloi, O.G. Adeyemi

**Affiliations:** a Department of Mathematical Sciences, College of Natural Sciences, Redeemer's University, Ede, Nigeria; b Department of Mathematics, University of Lagos, Akoka, Nigeria; c Department of Education, Vaal University of Technology, Vanderbijlpark 1911, South Africa; d Department of Chemical Sciences, College of Natural Sciences, Redeemer's University, Ede, Nigeria

**Keywords:** Applied mathematics, Computational mathematics, Thermodynamics

## Abstract

The present article investigates the entropy generation rate in the nonlinear convective flow of a reactive couple stress liquid through a channel filled with saturated materials and subjected to convective cooling. Analytical solutions of the coupled nonlinear boundary-value problems arising from the mathematical formulation are obtained by using the Homotopy Analysis Method (HAM). The analytical solutions are further validated numerically with the fourth order Runge-Kutta (RK4) to establish the accuracy of the method. Velocity, temperature, entropy generation, and heat irreversibility ratio profiles are presented and discussed extensively. The result of the computation shows that entropy generation increases significantly with increasing buoyancy parameter.

## 1 Introduction

Over the last few years, energy conversion and management has experienced a tremendous attention because of the need to reduce energy wastage. For this to be achievable, it is important to minimize energy loss from heat transfer and dissipation to boost the exergy of the thermal system. In view of this fact, a good number of researchers have been working on ways to enhance the performance of thermal systems based on the second law of thermodynamics method. At the forefront, of the study is Bejan [1, 2, 3] in which, thermodynamics laws are incorporated into equations governing fluid flow. Following his analysis, Sobamowo and Akinshilo [4] presented a perturbative approach to analyzing the entropy generation in a fourth-grade fluid. Torabi *et al.* [5] presented a review of the relevant work done on heat irreversibility analysis through flow passages with saturated the porous materials. Falade *et al.* [6] addressed the entropy production in a variable viscous couple stress fluid while Anand [7] studied the heat irreversibility in a tube with a nanofluid flow. Also, Zhu *et al.* [8] were concerned with the double diffusivity problem in a power-law fluid. Srinivasacharya and Bindu [9, 10] presented a result connected with heat irreversibility in annuli with micropolar fluid. More recently, Biswal and Basak [11] documented various techniques used in minimizing entropy in convective flow problems. Lopez de Haro *et al.* [12] reported the entropy generation in the power-law liquid in a channel with asymmetrical convective heating. Finally, Ibanez [13] examined the inherent heat irreversibility in a microchannel. For the sake of brevity, interested readers can see more interesting results in on thermodynamics analysis in [14, 15, 16, 17, 18] and the references contained.

With growing attention on reacting nonlinear convective flow, Qayyum *et al.* [19] discussed the nonlinear convective current in the developing flow of third-grade fluid undergoing a destructive chemical reaction. In the work of Hayat *et al.* [20], the electrically conducting Walters-B with nanoparticles over a stretching sheet with variable thickness was discussed extensively. Similarly, some nonlinear convective flows of hydromagnetic nanofluid were investigated using several constitutive models by Hayata and his collaborators. For instance, in [21] the Oldroyd-B model, Jeffry model [22] and thixotropic model in [23]. Also, Mahanthesh *et al.* [24] reported the nonlinear convection in the tangent hyperbolic fluid under the heated vertical channel. Qayyum *et al.* [25, 26] described the Eyring-Powel nanofluid and thixotropic fluid endowed with the Cattaneo-Christov heat flux condition respectively. Shaw *et al.* [27] analyzed the nonlinear convection in the flow of Casson fluid.

In the true sense, flow situations involving the strongly reactive fluid under Arrhenius kinetics in a vertical channel is expected to be more complex in terms of the heat transfer and dissipation and it is logical enough that the linearized Boussinesq approximation may not accurately determine the thermal structure. Therefore, the main objective of the present study is to extend the study in [28] to the nonlinear convection case with convective cooling at the walls. After an exhaustive literature survey, it is discovered that the study described here has not been addressed in spite of its important applications in oil recovery.

## 2 Analysis

**Fig. 1:**
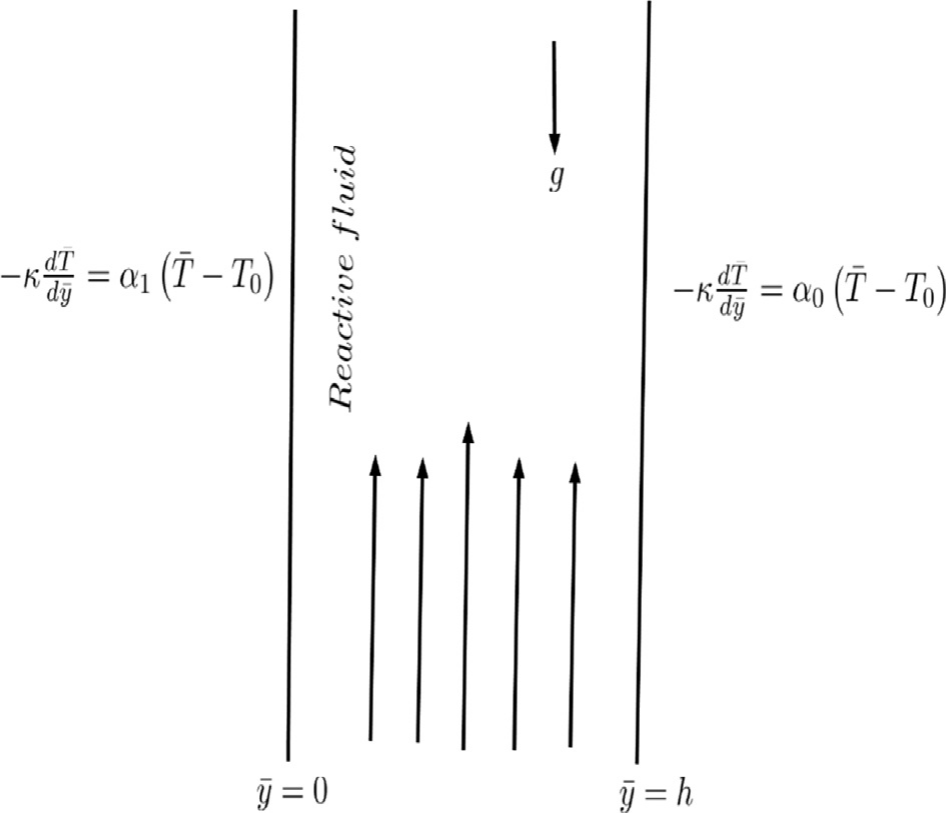
Geometry of the problem.

Consider the fully developed nonlinear convective flow of a reactive couple stress fluid through parallel vertical plates positioned at distance ‘*h*’ apart as presented in Fig. 1. The forced convective flow is induced by the combination of the constant pressure gradient and temperature difference. The channel under consideration is assumed to be saturated with porous materials and exchanges heat with the ambient in a pattern that follows Newtonâ€™s law of cooling. In view of these flow assumptions, the coupled governing equations are as follows [19, 20, 21, 22, 23, 24, 25, 26, 27, 28]:

(1) $$0=-\frac{dP}{dx}+\mu \frac{d^{2}\bar{u}}{d{\bar{y}}^{2}}-\mu \frac{\bar{u}}{K}-\eta \frac{d^{4}\bar{u}}{d{\bar{y}}^{4}}+\rho g\beta_{0}\left(\bar{T}-T_{0}\right)+\rho g\beta_{1}{\left(\bar{T}-T_{0}\right)}^{2}$$

(2) $$0=\frac{d^{2}\bar{T}}{d{\bar{y}}^{2}}+\frac{QC_{0}A}{\kappa }e^{-\frac{E}{R\bar{T}}}+\frac{\mu }{\kappa }{\left(\frac{d\bar{u}}{d\bar{y}}\right)}^{2}+\frac{\mu {\bar{u}}^{2}}{\kappa K}+\frac{\eta }{\kappa }{\left(\frac{d^{2}\bar{u}}{d{\bar{y}}^{2}}\right)}^{2}$$

subjected to the boundary conditions

(3) $$\begin{array}{l} \bar{u}=\frac{d^{2}\bar{u}}{d{\bar{y}}^{2}}=0,\;-\kappa \frac{d\bar{T}}{d\bar{y}}=\alpha_{0}\left(\bar{T}-T_{0}\right),\;\text{on}\;\bar{y}=h\\ \bar{u}=\frac{d^{2}\bar{u}}{d{\bar{y}}^{2}}=0,\;-\kappa \frac{d\bar{T}}{d\bar{y}}=\alpha_{1}\left(\bar{T}-T_{0}\right),\;\text{on}\;\bar{y}=0 \end{array}\}$$

The expression for the volumetric rate of entropy generation in the couple stress fluid flow can be expressed as:

(4) $$E_{G}=\frac{\kappa }{T_{0}^{2}}{\left(\frac{d\bar{T}}{d\bar{y}}\right)}^{2}+\frac{\mu }{T_{0}}{\left(\frac{d\bar{u}}{d\bar{y}}\right)}^{2}+\frac{\mu {\bar{u}}^{2}}{T_{0}K}\frac{\eta }{T_{0}}{\left(\frac{d{\bar{u}}^{2}}{d{\bar{y}}^{2}}\right)}^{2}$$

where P is the modified pressure, μ is the combustible material dynamic viscosity coefficient, $\bar{u}$ is the axial velocity, K is the porous media permeability, η is the fluid particle size effect due to couple stresses, ρ is the fluid density, g is acceleration due to gravity, $\beta_{0}$ is the linear thermal expansion coefficient, $\bar{T}$ is the absolute temperature, $T_{0}$ is the ambient temperature, $\beta_{1}$ is the nonlinear thermal expansion coefficient, Q is the heat of reaction, $C_{0}$ is the initial concentration of the species, A is the rate constant, κ is the thermal conductive, E is the activation energy, R is the universal gas constant, $\alpha_{0}$ and $\alpha_{1}$ are the convective heat transfer coefficients for the slab upper and lower surfaces respectively.

Introducing the following dimensionless variables in (5):

(5) $$y=\frac{\bar{y}}{h},\;u=\frac{\bar{u}}{UM},\;\theta =\frac{E\left(\bar{T}-T_{0}\right)}{RT_{0}^{2}}\;\text{and}\;M=-\frac{h^{2}}{\mu U}\frac{dP}{dx},$$

we arrive at the following ordinary differential equations together with the boundary conditions (1)–(3) yield

(6) $$1+\frac{d^{2}u}{dy^{2}}-\beta^{2}u-\frac{1}{\gamma }\frac{d^{4}u}{dy^{4}}+Gr\left(\theta +\sigma \theta^{2}\right)=0,\;\gamma \neq 0$$

(7) $$\frac{d^{2}\theta }{dy^{2}}+\lambda \left\{\exp\left(\frac{\theta }{1+\varepsilon \theta }\right)+\delta \left[{\left(\frac{du}{dy}\right)}^{2}+\frac{1}{\gamma }{\left(\frac{d^{2}u}{dy^{2}}\right)}^{2}+\beta^{2}u^{2}\right]\right\}=0,\;\gamma \neq 0$$

(8) $$u=\frac{d^{2}u}{dy^{2}}=0,\;\frac{d\theta }{dy}=-B_{0}\theta ,\;\text{on}\;y=0,$$

(9) $$u=\frac{d^{2}u}{dy^{2}}=0,\;\frac{d\theta }{dy}=-B_{1}\theta ,\;\text{on}\;y=1,$$

while the expression for the local entropy generation rate in Eq. (4) gives:

(10) $$Ns=\frac{E_{G}h^{2}E^{2}}{\kappa R^{2}T_{0}^{2}}={\left(\frac{d\theta }{dy}\right)}^{2}+\frac{\delta \lambda }{\varepsilon }\left({\left(\frac{du}{dy}\right)}^{2}+\frac{1}{\gamma }{\left(\frac{d^{2}u}{dy^{2}}\right)}^{2}+\beta^{2}u^{2}\right),\;\gamma \neq 0$$

to arrive at the above dimensionless Eqs. (6), (7), (8), (9), and (10), the following parameters have been used in the procedure

(11) $$\begin{array}{c} \beta =\sqrt{\frac{1}{Da}},\;Da=\frac{K}{h^{2}},\;\gamma =\frac{h}{l},\;l=\sqrt{\frac{\eta }{\mu }},\;Gr=\frac{h^{2}}{M\mu U}\frac{\rho g\beta_{0}RT_{0}^{2}}{\gamma^{2}E},\;\sigma =\frac{\beta_{1}RT_{0}^{2}}{\beta_{0}E},\\ \lambda =\frac{QEAC_{0}h^{2}}{RT_{0}^{2}\kappa }\exp\left(-\frac{E}{RT_{0}}\right),\;\varepsilon =\frac{RT_{0}}{E},\;\delta =\frac{U^{2}\mu M^{2}}{QAC_{0}h^{2}}\exp\left(\frac{E}{RT_{0}}\right),\;B_{0}=\frac{h\alpha_{0}}{\kappa },\;B_{1}=\frac{h\alpha_{1}}{\kappa } \end{array}$$

here in (11), β represents the porous medium permeability parameter, Da denotes the Darcy number, γ is the couple stress inverse parameter, l is the a function of molecular dimension of the fluid, Gr is the local Grashof number representing the effects of buoyancy in the flow system, σ stands for nonlinear thermal convection parameter, λ is the Frank - Kameneskii parameter, ε represents the activation energy parameter, δ is the viscous heating parameter, $B_{0}$ and $B_{1}$ stands for the Biot numbers for the slab lower and upper surfaces respectively.

From (10), if we set

(12) $$M_{1}={\left(\frac{d\theta }{dy}\right)}^{2},\;M_{2}=\frac{\delta \lambda }{\varepsilon }\left({\left(\frac{du}{dy}\right)}^{2}+\frac{1}{\gamma }{\left(\frac{d^{2}u}{dy^{2}}\right)}^{2}+\beta^{2}u^{2}\right),\;\gamma \neq 0$$

In (12), $M_{1}$ and $M_{2}$ are the irreversibility due to heat transfer and fluid friction respectively, then the Bejan number, Be that measures the ratio of heat transfer and fluid friction within the flow channel can be written as

(13) $$Be=\frac{M_{1}}{M_{1}+M_{2}}=\frac{1}{1+\varphi },\;\varphi =\frac{M_{2}}{M_{1}}.$$

From Eq. (13), it is worthwhile to known that the Bejan number (Be) ranges from 0≤Be≤1 and the following are valid

(14) $$Be=\{\begin{array}{ll} 0, & M_{2}>>M_{1};\\ 0.5, & M_{1}=M_{2};\\ 1, & M_{1}>>M_{2}. \end{array}$$

The parameter range given in (14) at Be = 0 represents the limiting case when viscous interaction dominated over heat transfer irreversibility and Be=1 corresponds to the limiting case when heat transfer irreversibility dominates absolutely while equal contributions from both heat transfer and fluid friction irreversibility to entropy production in the flow channel gives Be=0.5.

The system of coupled nonlinear differential Eqs. (6) and (7) subject to the boundary conditions of Eqs. (8) and (9) are solved analytically via the homotopy analysis method (HAM) as described in [29, 30]. To solve Eqs. (6) and (7), we choose the initial approximation for u and θ as follows:

(15) $$u_{0}=\frac{\gamma }{24}y\left(y-1\right)\left(y^{2}-y-1\right)\;\text{and}\;\theta_{0}=0$$

The solution (15) satisfies the zeroth order problem. The boundary conditions Eqs. (8) and (9) and the linear operators $L_{u}$ and $L_{\theta }$ are also defined as:

(16) $$L_{u}=\frac{d^{4}}{dy^{4}}\;\text{and}\;L_{\theta }=\frac{d^{2}}{dy^{2}}$$

with the properties

(17) $$L_{u}\left[c_{1}+c_{2}y+\frac{1}{2}c_{3}y^{2}+\frac{1}{6}c_{4}y^{3}\right]=0$$

(18) $$L_{\theta }\left[c_{5}+c_{6}y\right]=0$$

where $c_{i}\left(i=1..6\right)$ are the arbitrary integration constants determined from the boundary conditions. If p∈[0,1] denotes an embedding parameter, and $\hbar_{u}$ and $\hbar_{\theta }$ are the non-zero parameters, then the zeroth order deformation problems are:

(19) $$\left(1-p\right)L_{u}\left[\hat{u}\left(y;p\right)-u_{0}\left(y\right)\right]=p\hbar_{u}N_{1}\left[\hat{u}\left(y;p\right),\hat{\theta }\left(y;p\right)\left(y;p\right)\right]$$

(20) $$\left(1-p\right)L_{\theta }\left[\hat{\theta }\left(y;p\right)-\theta_{0}\left(y\right)\right]=p\hbar_{\theta }N_{2}\left[\hat{u}\left(y;p\right),\hat{\theta }\left(y;p\right)\left(y;p\right)\right]$$

subject to the boundary conditions

(21) $$\hat{u}\left(0;p\right)={\hat{u}}^{\prime \prime }\left(0;p\right)=0,\;{\hat{\theta }}^{\prime }\left(0;p\right)=-B_{0}\hat{\theta }\left(0;p\right),\;\hat{u}\left(1;p\right)={\hat{u}}^{\prime \prime }\left(1;p\right)=0,\;{\hat{\theta }}^{\prime }\left(1;p\right)=-B_{1}\hat{\theta }\left(1;p\right)$$

where $N_{1}$ and $N_{2}$ are the nonlinear operators defined as follows:

(22) $$N_{1}\left[\hat{u}\left(y;p\right),\hat{\theta }\left(y;p\right)\right]=1+\frac{\partial^{2}\hat{u}\left(y;p\right)}{\partial y^{2}}-\beta^{2}\hat{u}\left(y;p\right)-\frac{1}{\gamma }\frac{\partial^{4}\hat{u}\left(y;p\right)}{\partial y^{4}}+Gr\hat{\theta }\left(y;p\right)(1+\sigma \hat{\theta }\left(y;p\right)),\;\gamma \neq 0$$

(23) $$N_{2}\left[\hat{u}\left(y;p\right),\hat{\theta }\left(y;p\right)\right]=\frac{\partial^{2}\hat{\theta }\left(y;p\right)}{\partial y^{2}}+\lambda \left\{\exp\left(\frac{\hat{\theta }\left(y;p\right)}{1+\varepsilon \hat{\theta }\left(y;p\right)}\right)+\delta \left[{\left(\frac{\partial \hat{u}\left(y;p\right)}{\partial y}\right)}^{2}+\frac{1}{\gamma }{\left(\frac{\partial^{2}\hat{u}\left(y;p\right)}{\partial y^{2}}\right)}^{2}+\beta^{2}\hat{u}{\left(y;p\right)}^{2}\right]\right\}.$$

For *p* = 0 and *p* = 1 we have

(24) $$\hat{u}\left(y;0\right)=u_{0}\left(y\right),\;\hat{\theta }\left(y;0\right)=\theta_{0}\left(y\right),\;\hat{u}\left(y;1\right)=u\left(y\right),\;\hat{\theta }\left(y;1\right)=\theta \left(y\right),$$

and when *p* variation is taken from 0 to 1 then u(y;p) and θ(y;p) approach $u_{0}\left(y\right)$ and $\theta_{0}\left(y\right)$ to u(y) and θ(y). Now expanding u(y;p) and θ(y;p) in Taylor's series with respect to p yields the following:

(25) $$u\left(y;p\right)=u_{0}\left(y\right)+\sum_{n=1}^{\infty }u_{n}\left(y\right)p^{n}$$

(26) $$\theta \left(y;p\right)=\theta_{0}\left(y\right)+\sum_{n=1}^{\infty }\theta_{n}\left(y\right)p^{n}$$

where

(27) $$u_{n}\left(y\right)={\frac{1}{n!}\frac{\partial^{n}u\left(y;p\right)}{\partial p^{n}}|}_{p=0}\;\text{and}\;\theta_{n}\left(y\right)={\frac{1}{n!}\frac{\partial^{n}\theta \left(y;p\right)}{\partial p^{n}}|}_{p=0}.$$

By proper choice of the auxiliary linear operators, initial guesses and auxiliary parameters, the series Eqs. (25) and (26) converge for *p* = 1 and hence

(28) $$u\left(y\right)=u_{0}\left(y\right)+\sum_{n=1}^{\infty }u_{n}\left(y\right).$$

(29) $$\theta \left(y\right)=\theta_{0}\left(y\right)+\sum_{n=1}^{\infty }\theta_{n}\left(y\right).$$

The *n*th order deformation problems are:

(30) $$L_{u}\left[u_{n}\left(y\right)-\chi_{n}u_{n-1}\left(y\right)\right]=\hbar_{u}R_{u}^{n}\left(y\right)$$

(31) $$L_{\theta }\left[\theta_{n}\left(y\right)-\chi_{n}\theta_{n-1}\left(y\right)\right]=\hbar_{\theta }R_{\theta }^{n}\left(y\right)$$

subject to the boundary conditions:

(32) $$u_{n}\left(0\right)=u_{n}^{\prime \prime }\left(0\right)=u_{n}\left(1\right)=u_{n}^{\prime \prime }\left(1\right)=0,\;\theta_{n}^{\prime }\left(0\right)+B_{0}\theta_{n}\left(0\right)=0,\;\theta_{n}^{\prime }\left(1\right)+B_{1}\theta_{n}\left(1\right)=0,$$

and

(33) $$R_{u}^{n}\left(y\right)=\left(1-\chi_{n}\right)+u_{n-1}^{\prime \prime }-\beta^{2}u_{n-1}-\frac{1}{\gamma }u_{n}^{iv}+Gr\theta_{n-1}+\sigma \sum_{k=0}^{n-1}\theta_{n-1-k}\theta_{k},\;\gamma \neq 0$$

(34) $$R_{\theta }^{n}\left(y\right)=\theta_{n-1}^{\prime \prime }+\lambda \sum_{k}^{n-1}\left(1-\frac{k}{n}\right)\theta_{n-k}\Phi_{k}+\lambda \delta \left(\sum_{k=0}^{n-1}u_{n-1-k}^{\prime }u_{k}^{\prime }+\frac{1}{\gamma }\sum_{k=0}^{n-1}u_{n-1-k}^{\prime \prime }u_{}^{\prime \prime }+\beta^{2}\sum_{k=0}^{n-1}u_{n-1-k}u_{k}\right)$$

where

(35) $$\chi_{n}=\{\begin{array}{l} 0,n\leq 1;\\ 1,n>1. \end{array},\;\text{and}\;\Phi_{0}=\exp\left(\frac{\theta_{0}}{1+\varepsilon \theta_{0}}\right)$$

The general solution of equations are given by:

(36) $$u_{n}\left(y\right)=u_{n}^{*}\left(y\right)+c_{1}+c_{2}y+\frac{1}{2}c_{3}y^{2}+\frac{1}{6}c_{4}y^{3}$$

(37) $$\theta_{n}\left(y\right)=\theta_{n}^{*}\left(y\right)+c_{5}+c_{6}y$$

where $u_{n}^{*}$ and $\theta_{n}^{*}$ are the particular solutions. Constants $c_{i}\left(i=1\ldots 6\right)$ are determined by the boundary conditions Eq. (32).

Next, Eqs. (15), (16), (17), (18), (19), (20), (21), (22), (23), (24), (25), (26), (27), (28), (29), (30), (31), (32), (33), (34), (35), (36), and (37) are coded in a symbolic packages such as MATHEMATICA or MAPLE. Then Eqs. (30) and (31) can be solved one after the other in the order n=1,2,3,…. All computational work in this present study has been carried out by utilizing symbolic software MAPLE 18, running on an intel fifth-generation computer of 6G RAM.

## 3 Results and discussion

**Fig. 2:**
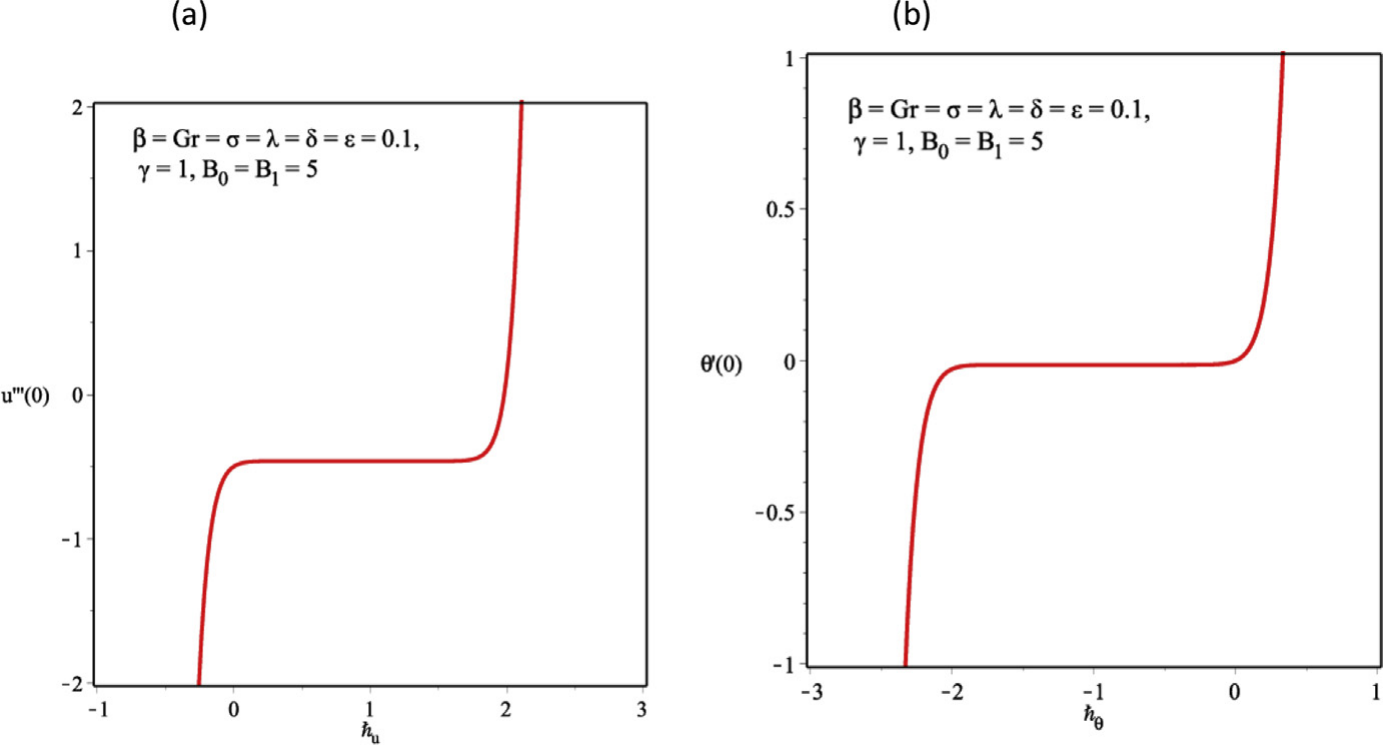
The ℏ-curve for the functions (a) u(y) (b) θ(y).

This section is dedicated to the discussion of the graphical results. Table 1 confirms the convergence of the solution obtained via HAM as the order of approximation increases. From the tabular result, convergence is achieved from the 10th order of approximation while Table 2 presents the validation of the HAM solution by using the RK4 method. From the tabular result, a good agreement is seen between the two results, therefore, the computed result is reliable. Fig. 2a, b represents the influence of the auxiliary parameters on the convergence of solutions and the admissible range for the auxiliary parameters are clearly shown in the plots. Obviously, from these figures the admissible range for $\hbar_{u}$ and $\hbar_{\theta }$ is $0\leq \hbar_{u}\leq 1.8$ and $-1.8\leq \hbar_{\theta }\leq -0.16$ respectively.

**Table 1:** Convergence of HAM solution for different order of approximation when $\beta =Gr=\sigma =\lambda =\delta =\varepsilon =\text{0.1},\gamma =\text{1},B_{\text{0}}=B_{\text{1}}=\text{5},\hbar_{u}=\text{0.7}$ and $\hbar_{\theta }=-\text{1.2}$.

Order of approximations	$u^{'''}\left(1\right)$	$-\theta^{'}\left(1\right)$
1	0.470804	0.036033
5	0.462469	0.030331
10	0.462445	0.030324
15	0.462445	0.030324
20	0.462445	0.030324
25	0.462445	0.030324
30	0.462445	0.030324

**Table 2:** Validation of solution when $\beta =Gr=\sigma =\lambda =\delta =\varepsilon =\text{0.1},\gamma =\text{1},B_{\text{0}}=B_{\text{1}}=\text{5},\hbar_{u}=\text{0.7}$ and $\hbar_{\theta }=-\text{1.2}$ at 15^th^*order*.

	u			θ		
y	HAM	RK4	Error	HAM	RK4	Error
0.0	0.0000000	0.0000000	0	−0.0140393	−0.0140391	2.20000000E-07
0.1	0.0037171	0.0037171	0	−0.0075138	−0.0075138	3.00000000E-08
0.2	0.0070292	0.0070292	0	−0.0019811	−0.0019812	1.10000000E-07
0.3	0.0096192	0.0096192	0	0.0025530	0.0025528	2.00000000E-07
0.4	0.0112624	0.0112624	0	0.0060837	0.0060834	3.20000000E-07
0.5	0.0118252	0.0118252	0	0.0086071	0.0086068	3.40000000E-07
0.6	0.0112629	0.0112629	0	0.0101207	0.0101204	3.70000000E-07
0.7	0.0096199	0.0096199	0	0.0106231	0.0106227	4.70000000E-07
0.8	0.0070300	0.0070300	0	0.0101139	0.0101134	5.00000000E-07
0.9	0.0037176	0.0037176	0	0.0085939	0.0085935	4.20000000E-07
1.0	0.0000000	0.0000000	0	0.0060651	0.0060647	3.90000000E-07

**Fig. 3:**
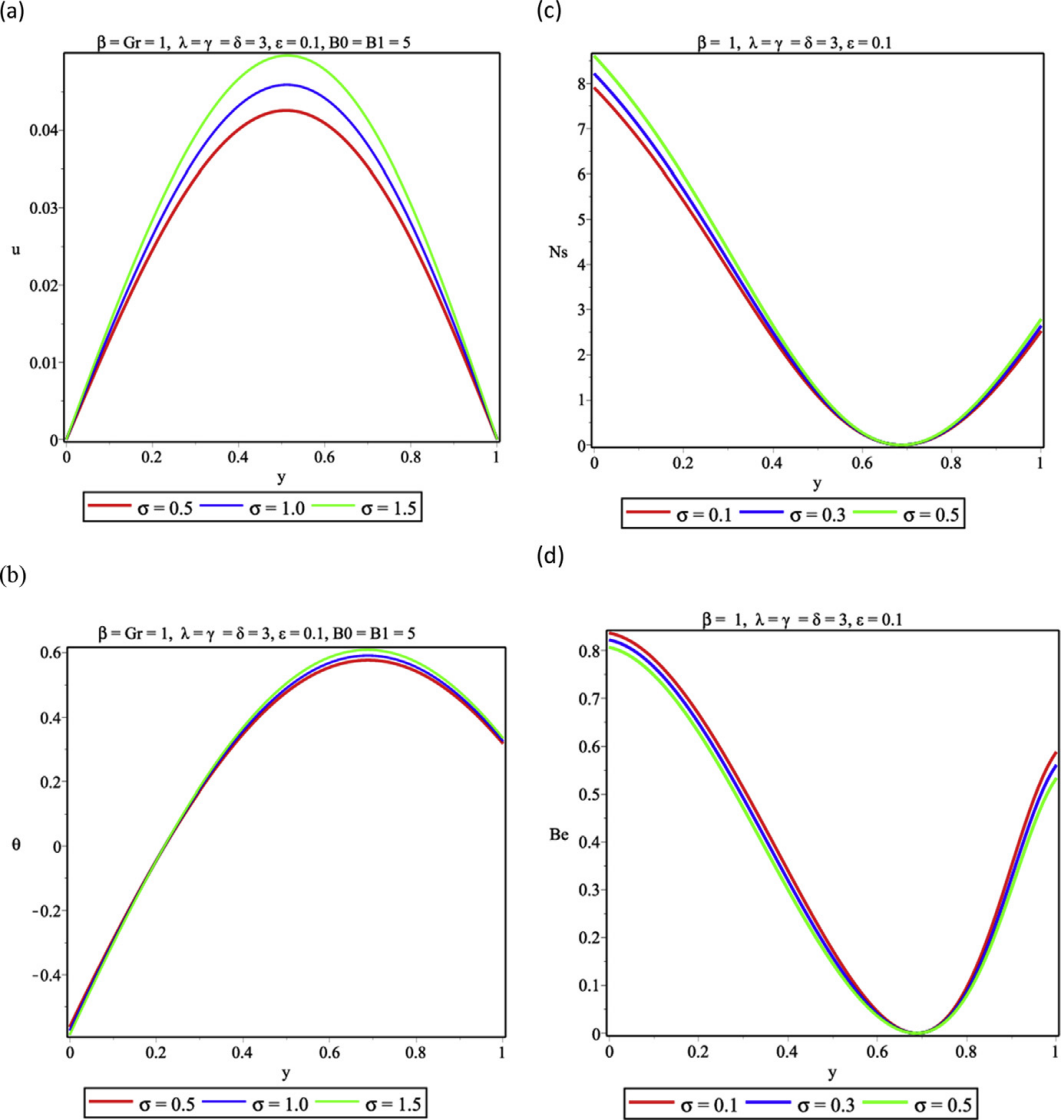
Effect of nonlinear convection (σ) (a) Velocity profile (b) Temperature profile (c) Entropy generation profile (d) Bejan number profile.

In Fig. 3, variations of the nonlinear convection term are presented. The variation of the nonlinear convection term gives rise to increased flow velocity as shown in Fig. 3a due to increase in the fluid temperature as the nonlinear convection parameter rises (Fig. 3b) since the fluid velocity and temperature rises with increasing nonlinear convection term. Entropy generation in the channel is expected to be on the increase accordingly as reported in Fig. 3c. As a result, HTI dominates over FFI throughout the porous medium except as some regions in the centerline of the channel where FFI controls the heat irreversibility (Fig. 3d).

**Fig. 4:**
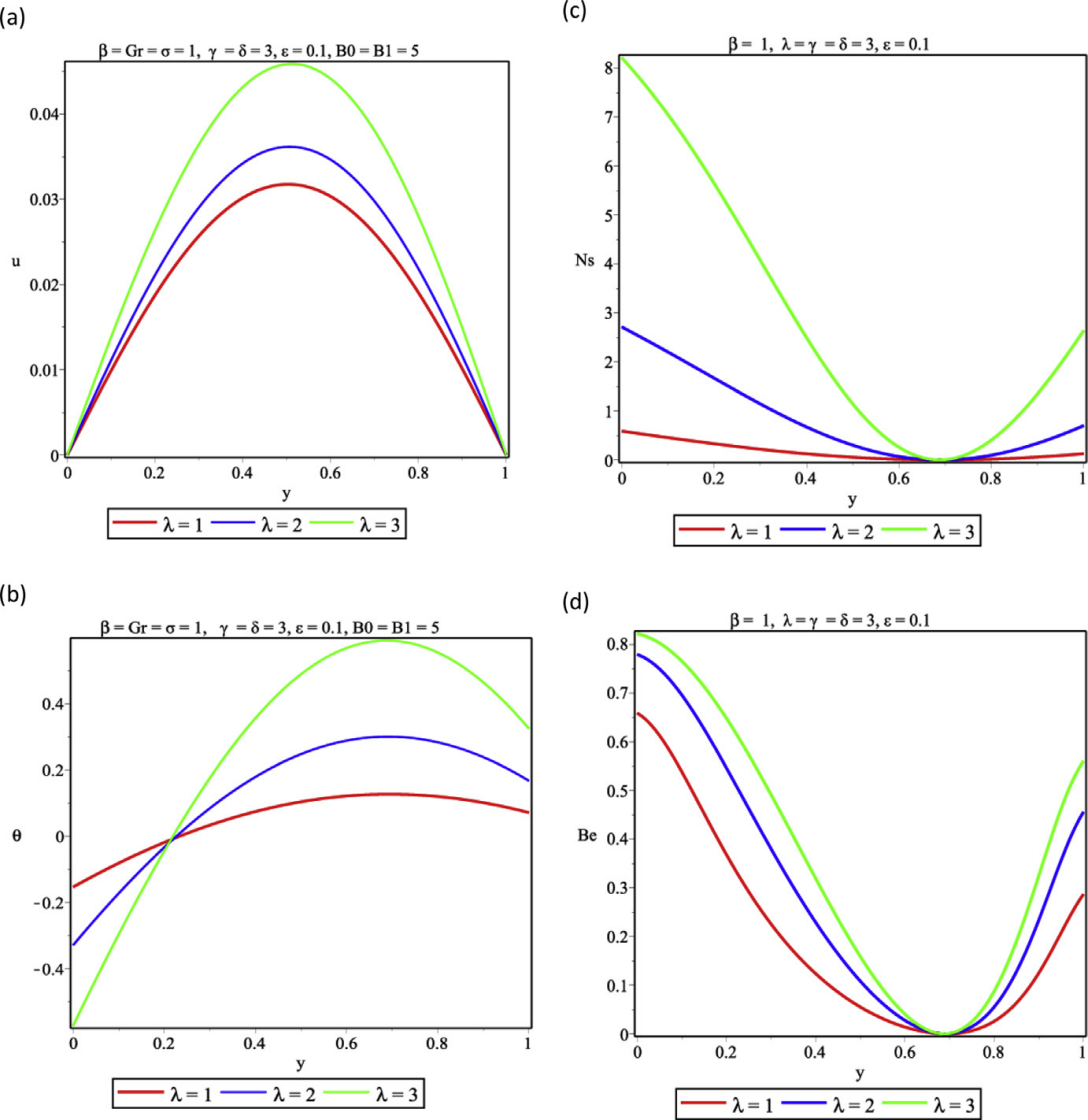
Effect of Frank-Kameneskii parameter (λ) (a) Velocity profile (b) Temperature profile (c) Entropy generation profile (d) Bejan number profile.

**Fig. 5:**
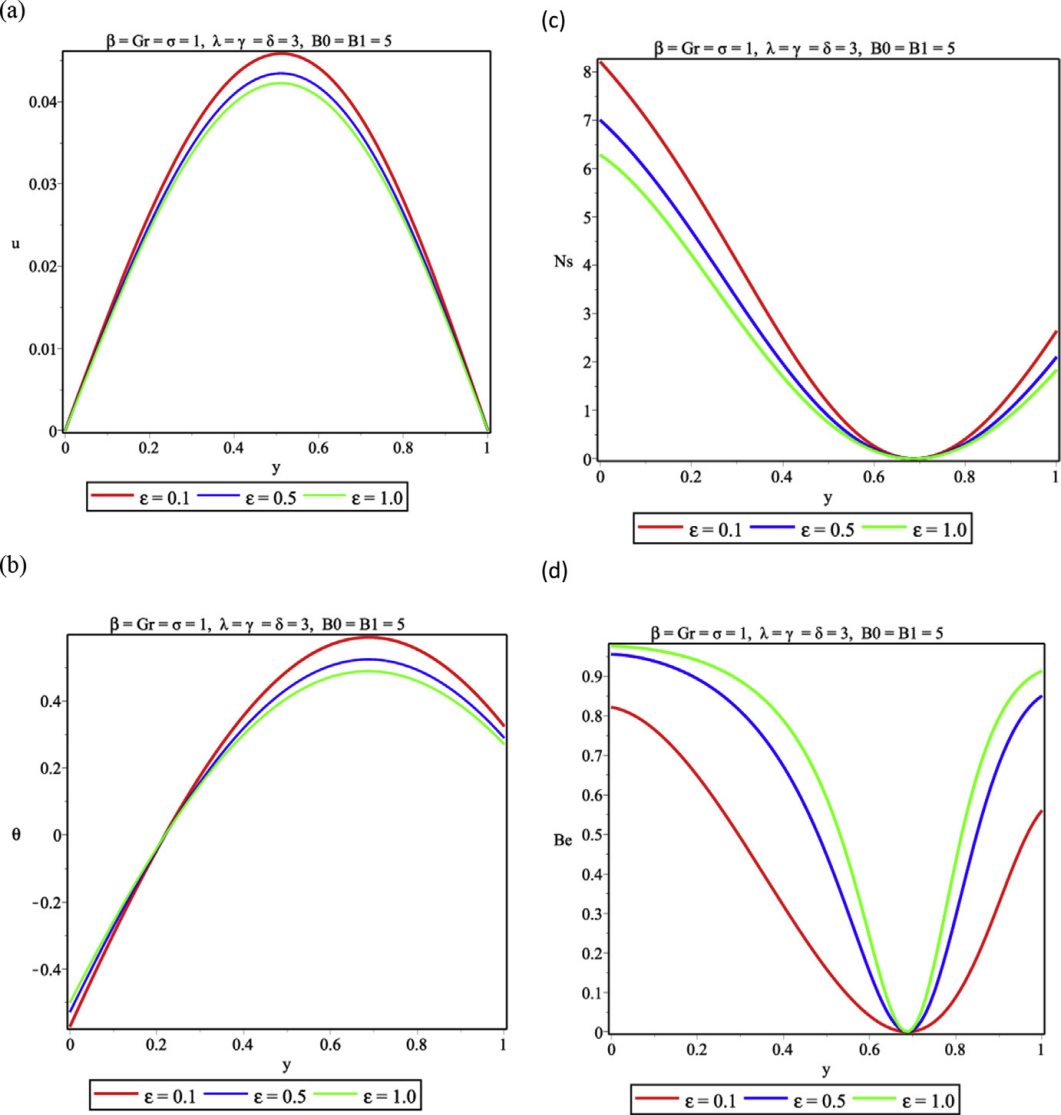
Effect of activation energy parameter (ε) (a) Velocity profile (b) Temperature profile (c) Entropy generation profile (d) Bejan number profile.

One important parameter in combustion is the Frank-Kameneskii (FK) parameter which arises from the Arrhenius kinetics of the exothermic couple stress fluid. The result from Fig. 4a shows that that the flow velocity increases with increasing values of the FK parameter, elevates the temperature distribution (Fig. 4b), promotes entropy generation (Fig. 4c) and supports the dominance of HTI over FFI in the flow channel (Fig. 4d). In Fig. 5, a variation of activation energy parameter is presented. As shown, as the activation energy parameter increases, there is a decrease in both fluid velocity and temperature as seen in Fig. 5a,b. This is so because of the decrease in the activation energy of the reactive liquid thus lowering the entropy generation rate. As a result, entropy decreases and HTI dominates over FFI in the vertical channel as seen in Fig. 5c and d respectively.

**Fig. 6:**
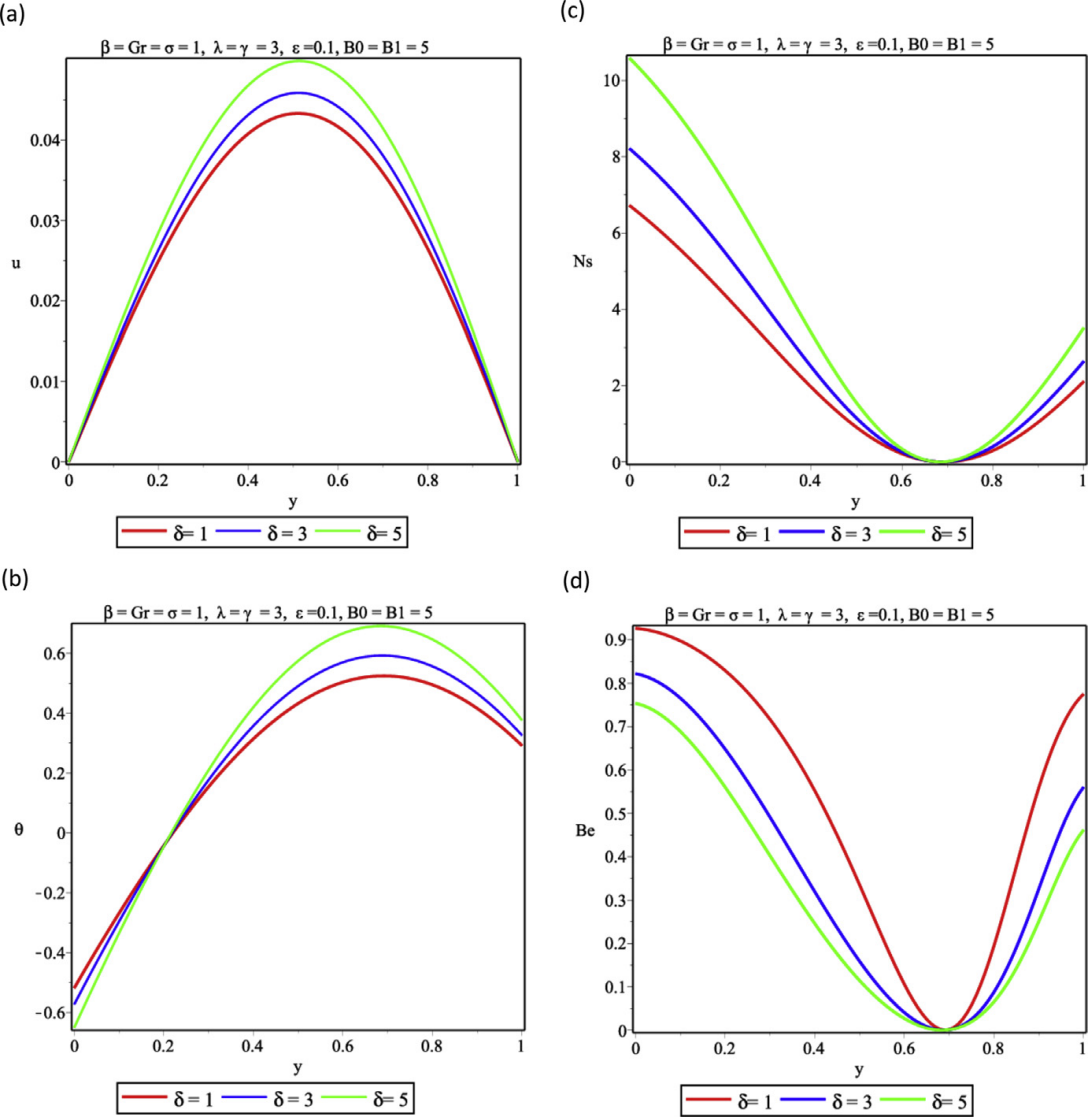
Effect of viscous dissipation (δ) (a) Velocity profile (b) Temperature profile (c) Entropy generation profile (d) Bejan number profile.

**Fig. 7:**
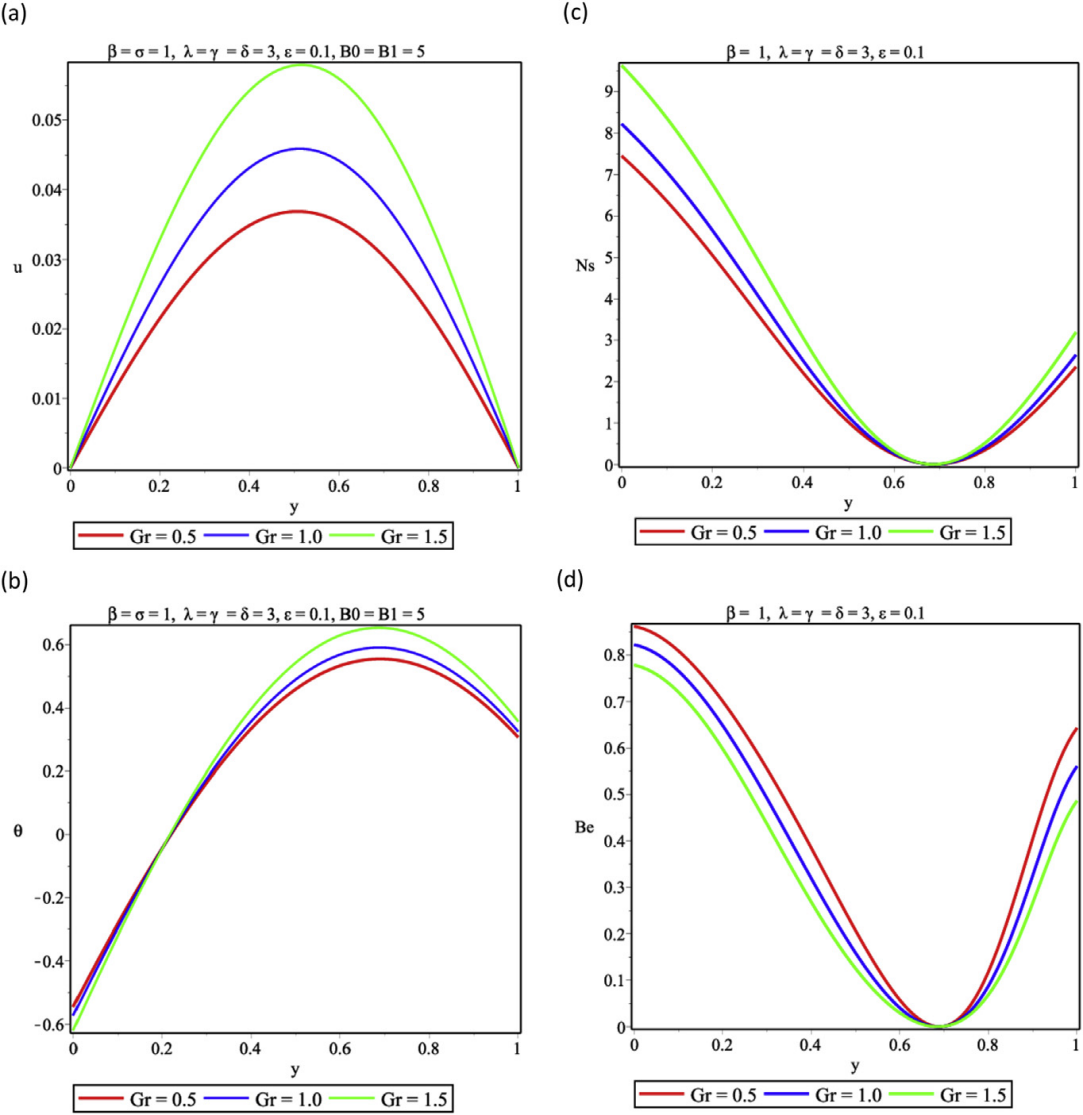
Effect of Grashof number (Gr) (a) Velocity profile (b) Temperature profile (c) Entropy generation profile (d) Bejan number profile.

**Fig. 8:**
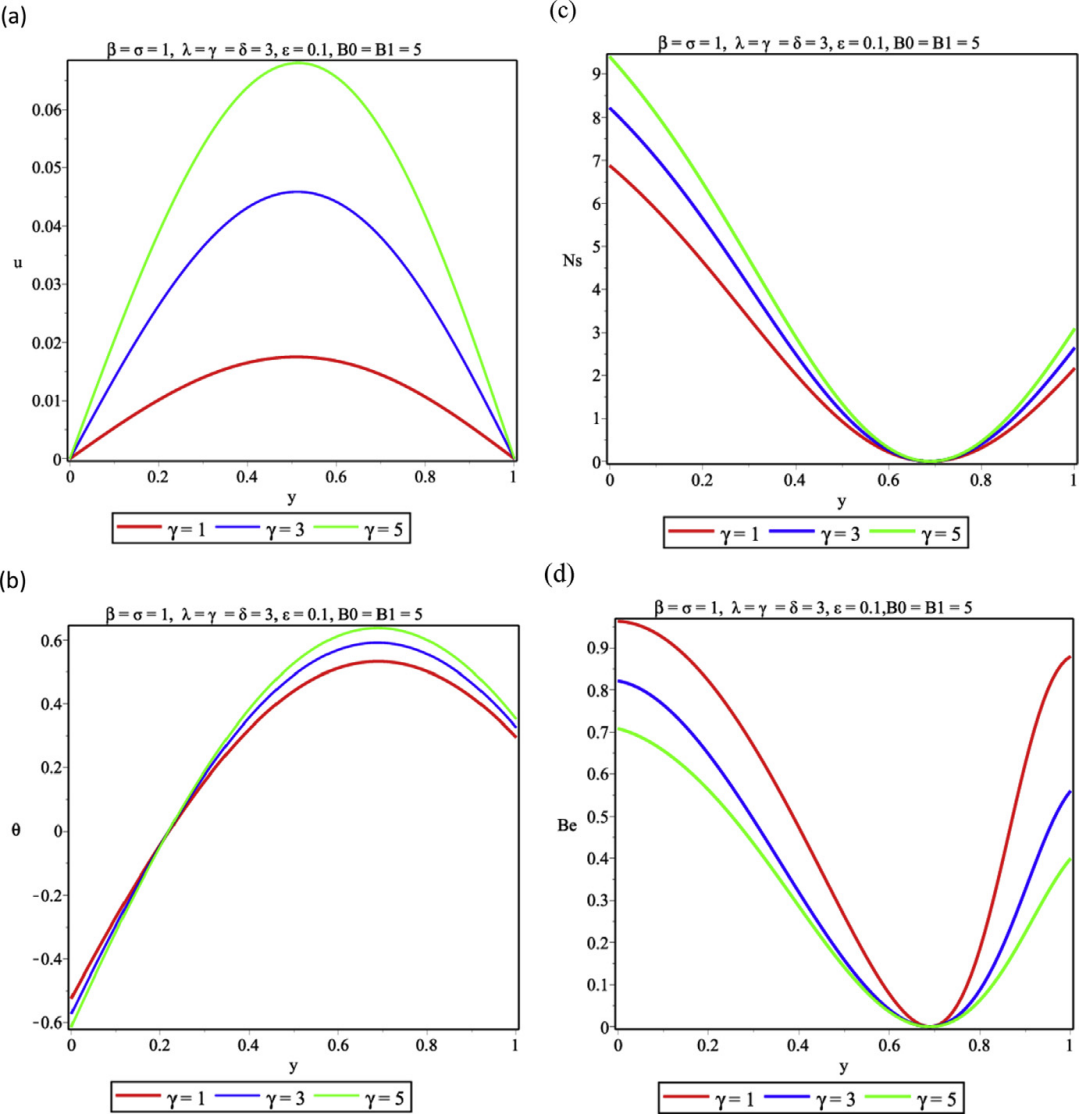
Effect of couple stress inverse parameter (γ) (a) Velocity profile (b) Temperature profile (c) Entropy generation profile (d) Bejan number profile.

The influence of viscous dissipation on the flow and thermal structure is presented in Fig. 6. Increasing the magnitude of the viscous dissipation parameter is seen in Fig. 6a to enhance the flow velocity significantly as a result of the decrease in the frictional forces in the layers of the fluid. Again energy is dissipated in form of heat as the exothermic reaction continues, this is shown in Fig. 6b and the entropy generation rises. As reported in Fig. 6c, heat released from viscous dissipation is a major factor in entropy generation analysis, therefore, as witnessed in Fig. 6d, heat transfer irreversibility plays a dominant role over that generated from viscous dissipation at the two walls. But as the viscous dissipation parameter increases FFI begins to show effects of the heat irreversibility ratio. Fig. 7 revealed the effect of variations in the fluid Grashof number on the buoyancy induced- flow, it is shown that by increasing the values of Grashof number enhances the flow and thermal distributions in Fig. 7 a,b respectively. This implies that entropy generation would be higher as shown in Fig. 7 c as a result of the heat transfer to the core region of the fluid, therefore HTI is seen to be significant at the walls except for the core region where FFI dominates completely as presented in Fig. 7d. Fig. 8 represents the response to variation of couple stress inverse parameter. Fig. 8a shows that the velocity of the fluid increases as the couple stress inverse parameter increases. This means that fluid thinning encourages the fluid flow, however, as the fluid thickens the reverse trend is expected. The thinning property also encourages temperature rise in Fig. 8b. This leads to energy loss and the entropy generation increases accordingly as seen in Fig. 8c. It is important to note that fluid thickening encourages energy conservation. Result in Fig. 8d shows that HTI dominates over FFI in the couple stress fluid flow in the porous medium.

## 4 Conclusion

The nonlinear convective flow of the reactive couple stress fluid with convective wall temperature had been investigated, the coupled nonlinear boundary value problems from the equations governing the fluid flow have been tackled numerically and analytically. Tables 1 and 2 have been used to establish the convergence of the HAM with increasing order of approximation increases. The result showed a perfect match as presented in Table 2. Summarily, for convective flow of exothermic couple stress fluid flowing through a vertical channel filled with porous materials, we claim that: increasing values of both linear and nonlinear convective parameter enhances the velocity profile, elevates the fluid temperature and depletes the energy of the system.

## Declarations

### Author contribution statement

Samuel O. Adesanya: Conceived and designed the analysis; Analyzed and interpreted the data; Contributed analysis tools or data; Wrote the paper.

Hammed A. Ogunseye, Kholeka C. Moloi: Conceived and designed the analysis; Analyzed and interpreted the data; Contributed analysis tools or data.

Ramosheuw S. Lebelo: Conceived and designed the analysis; Analyzed and interpreted the data.

Olalere G. Adeyemi: Analyzed and interpreted the data; Contributed analysis tools or data.

### Funding statement

This research did not receive any specific grant from funding agencies in the public, commercial, or not-for-profit sectors.

### Competing interest statement

The authors declare no conflict of interest.

### Additional information

No additional information is available for this paper.
